# Runx1 stabilizes the mammary epithelial cell phenotype and prevents epithelial to mesenchymal transition

**DOI:** 10.18632/oncotarget.15381

**Published:** 2017-02-16

**Authors:** Deli Hong, Terri L. Messier, Coralee E. Tye, Jason R. Dobson, Andrew J. Fritz, Kenneth R. Sikora, Gillian Browne, Janet L. Stein, Jane B. Lian, Gary S. Stein

**Affiliations:** ^1^ Department of Biochemistry and University of Vermont Cancer Center, University of Vermont College of Medicine, Burlington, VT, USA; ^2^ Department of Cell and Developmental Biology, University of Massachusetts Medical School, Worcester, MA, USA

**Keywords:** Runx1, epithelial to mesenchymal transition, epithelial Integrity, patient survival, breast cancer

## Abstract

Runx1 is a well characterized transcription factor essential for hematopoietic differentiation and Runx1 mutations are the cause of leukemias. Runx1 is highly expressed in normal epithelium of most glands and recently has been associated with solid tumors. Notably, the function of Runx1 in the mammary gland and how it is involved in initiation and progression of breast cancer is still unclear. Here we demonstrate the consequences of Runx1 loss in normal mammary epithelial and breast cancer cells. We first observed that Runx1 is decreased in tumorigenic and metastatic breast cancer cells. We also observed loss of Runx1 expression upon induction of epithelial-mesenchymal transition (EMT) in MCF10A (normal-like) cells. Furthermore depletion of Runx1 in MCF10A cells resulted in striking changes in cell shape, leading to mesenchymal cell morphology. The epithelial phenotype could be restored in breast cancer cells by re-expressing Runx1. Analyses of breast tumors and patient data revealed that low Runx1 expression is associated with poor prognosis and decreased survival. We addressed mechanisms for the function of Runx1 in maintaining the epithelial phenotype and find Runx1 directly regulates E-cadherin; and serves as a downstream transcription factor mediating TGFβ signaling. We also observed through global gene expression profiling of growth factor depleted cells that induction of EMT and loss of Runx1 is associated with activation of TGFβ and WNT pathways. Thus these findings have identified a novel function for Runx1 in sustaining normal epithelial morphology and preventing EMT and suggest Runx1 levels could be a prognostic indicator of tumor progression.

## INTRODUCTION

Evidence is rapidly accruing for the oncogenic and tumor suppressor functions of the Runx family of transcription factors, Runx1, Runx2 and Runx3, which are essential for normal lineage specific development [1, 2]. In late stage cancer, including breast, prostate and thyroid, abnormal expression of Runx2 drives metastasis to bone [3–5]. Inhibition of Runx2 in metastatic breast and prostate cancer cells drastically reduces tumor growth and metastasis *in vivo* [3, 6], revealing Runx2 function as an oncogene. It has been well documented that translocations of Runx1, the essential hematopoiesis factor, with ETO, TEL (ETV6) [7] or other genes cause a wide range of leukemias [8]. However, little is known of Runx1 oncogenic or tumor suppressor activities in solid tumors. An early microarray profiling study comparing adenocarcinoma metastasis with primary adenocarcinoma tumors identified Runx1 as one of 17 genes signature that associate with metastasis [9]. Recent genetic studies have identified loss-of-function somatic mutations or deletion of Runx1 in breast cancer patients [10, 11]. These data are consistent with evidence that Runx1 is reduced in metastasis-prone solid tumors [9]. There is a requirement for understanding Runx1-mediated regulatory mechanism(s) in breast cancer.

Breast cancer remains the leading cause of cancer related death in women worldwide [12]. Among the different subtypes of breast cancer, both the basal-like and Her2-enriched subtypes are the most clinically challenging; they have the worst survival rates and are often associated with metastasis [13]. It has been speculated that this aggressive phenotype of basal like breast cancer is linked with the Epithelial to Mesenchymal Transition (EMT), which is a key biological process in cancer progression and is involved in the conversion of early stage tumors into invasive malignancies [14]. Oncogenic EMT occurs when primary tumor cells undergo a switch from an epithelial phenotype, which lacks motility and exhibits extensive cell-to-cell contact, to a mesenchymal phenotype having high cellular motility, lower cellular interactions, and a non-polarized cell organization [15]. Several studies, using breast cancer cell lines and clinical samples, have demonstrated that increased expression of mesenchymal markers including Vimentin, Fibronectin and N-cadherin, as well as reduced expression of epithelial markers including E-cadherin are observed in basal subtype breast cancer [8–11]. The specific mechanisms that preserve the structural and functional properties of the epithelial cells of the glandular tissues and protect normal epithelial cells from transitioning to malignancy in basal like breast cancer are compelling unresolved questions. We therefore have focused our studies on the functional activities of Runx1 in basal subtype breast cancer cells.

In this study, we hypothesize that Runx1 maintains the normal epithelial phenotype and that loss of Runx1 promotes EMT. Our results demonstrate that depletion of Runx1 in mammary epithelial cells disrupts/alters cellular morphology and suppress E-cadherin expression. We find that Runx1 level decreases during both TGFβ-induced and growth factor-starvation induced EMT, supporting a crucial role for Runx1 in preventing EMT. Furthermore our analysis of breast tumors and survival data supports the above finding that loss of Runx1 promotes tumor progression. Thus, these studies demonstrate that Runx1 functions to preserve epithelial phenotype in mammary epithelial cells and reveal that Runx1 has tumor suppressor potential in breast cancer.

## RESULTS

### Runx1 expression is decreased in breast cancer

**Figure 1:**
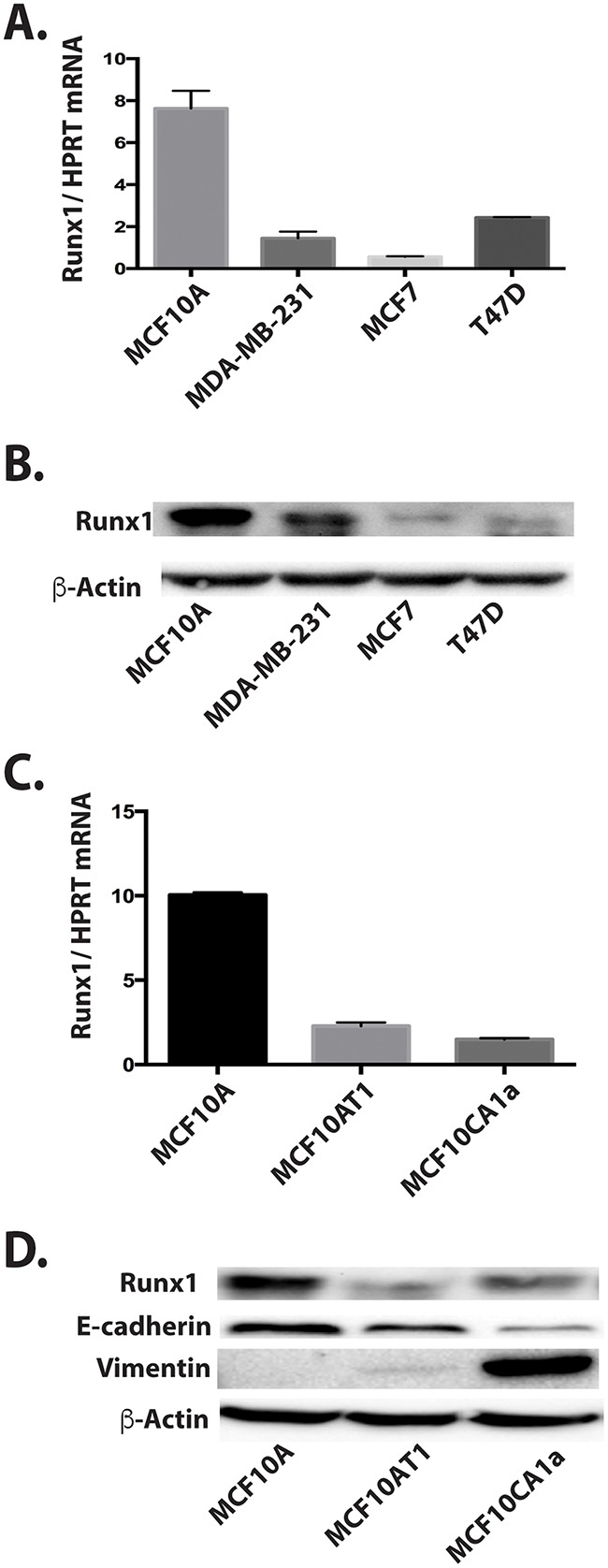
Decreased Runx1 expression is related to breast cancer progression in cell models **A**. Runx1 RNA expression by RT-qPCR for a panel of breast cancer cell lines compared to MCF10A cells show that Runx1 protein is decreased in breast cancer cells. **B**. Western blot of cell lysate for the same panel of cell lines shown in **A**. **C**. Runx1 RNA expression by RT-qPCR of normal mammary-like MCF10A cells, MCF10A-derived tumorigenic cell line MCF10AT1, and metastatic MCF10CA1a cells shows Runx1 is decreased in the cancer cells. **D**. Western blot comparison in the MCF10 series.

Runx1 involvement in breast cancer was first tested using a panel of normal and breast cancer cell lines representing different breast cancer subtypes (Figure 1). The selected cell lines included non-metastatic luminal-like MCF7 and T47D breast cancer cells and basal-like breast cancer MDA-MB-231 cells. Compared to the high level of Runx1 in normal-like basal MCF10A control cells, Runx1 mRNA (Figure 1A) and protein (Figure 1B) were significantly decreased in all breast cancer cell lines tested, but less so in the triple negative MDA-MB-231 cells.

We next evaluated Runx1 mRNA and protein expression in the MCF10 progression series of MCF10A normal-like mammary epithelial cells, tumorigenic MCF10AT1 and MCF10CA1a cells [16]. Runx1 mRNA (Figure 1C) and protein (Figure 1D) expression were strikingly decreased in both MCF10AT1 and MCF10CA1a cells compared with MCF10A cells. In both early and aggressive cancer cell types, loss of Runx1 expression paralleled decreases of the epithelial marker E-cadherin, while the mesenchymal marker Vimentin was highly expressed only in the MCF10CA1a cells. These changes in EMT markers are consistent with the mesenchymal phenotype of the two cancer cell lines. Thus decreased Runx1 with tumor progression correlates with EMT. Together our findings indicate an important role for Runx1 in normal breast epithelial cells and provide evidence for the emerging concept that Runx1 may function as a tumor suppressor [17].

### TGFβ induced EMT decreases Runx1 expression in MCF10A cells

The above results show that Runx1 levels are decreased in breast cancer cells and that decreased Runx1 is accompanied with EMT in the MCF10 series. To mechanistically address if decreased Runx1 and EMT are coupled in breast cancer, we used a well-known method to induce EMT in mammary cells, by adding TGFβ to MCF10A cells [18]. TGFB1-Smad signaling is the most frequently described inducer of EMT, and Runx1 is known to be a downstream target of TGFβ signaling. Furthermore it is well documented that Runx1 forms an interaction complex with SMADs [19], thereby regulating genes responsive to TGFβ. Taken together, we hypothesized that Runx1 expression would be repressed upon treating with TGFβ.

**Figure 2:**
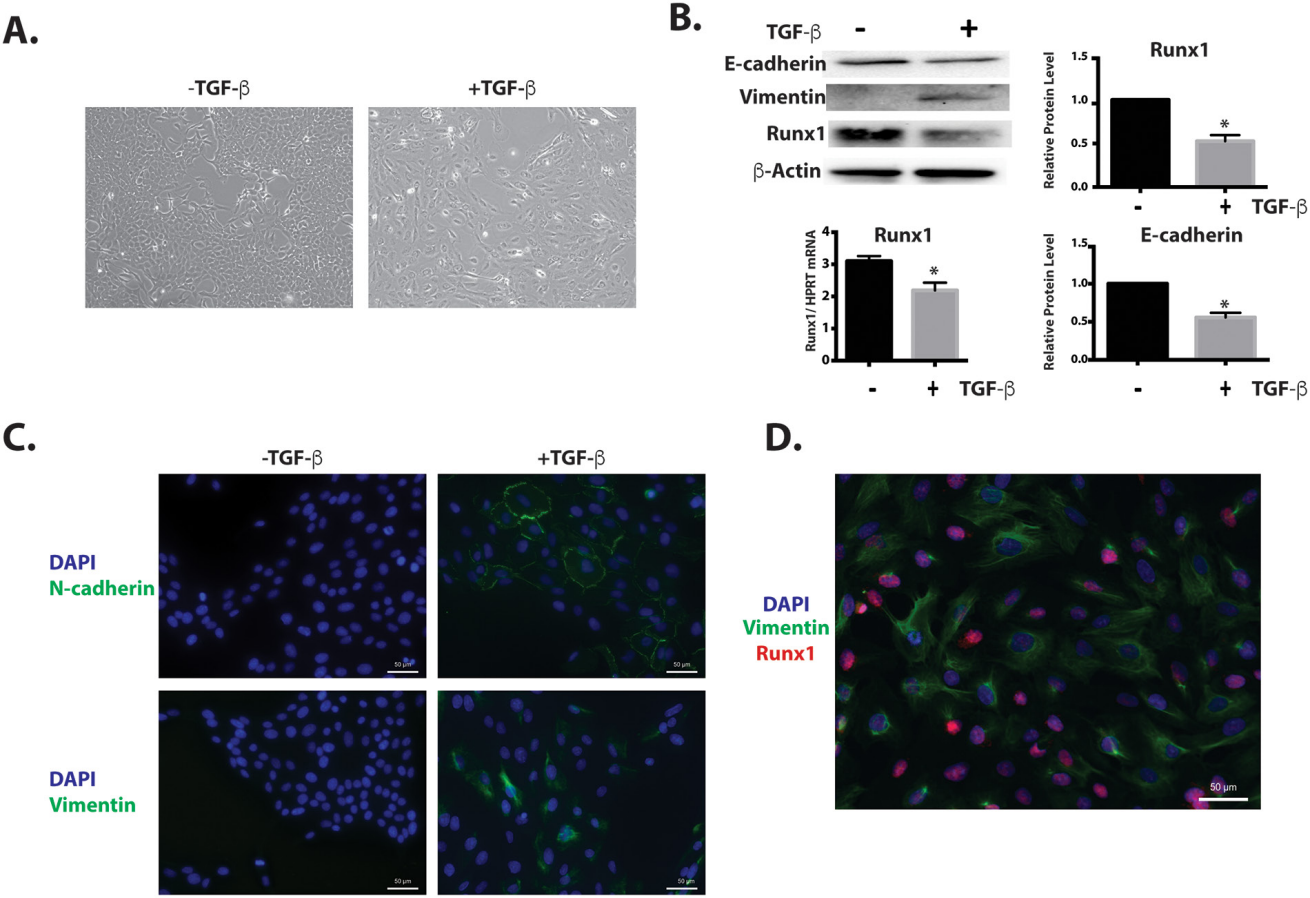
Runx1 decreases during TGFβ-induced EMT MCF10A cells treated with 10 ng/ml TGFβ for 6 days. **A**. MCF10A cells treated with TGFβ show morphological changes toward an EMT-like state. **B**. Western blot analyses show changes in EMT markers and Runx1 expression during EMT. Left lower panel: RT-qPCR of RNA from MCF10A cells shows decreased Runx1 expression in TGFβ treated cells. Student's *t* test * *p* value <0.05 for TGFβ-treated cells compared to control cells. Where error bars are shown these represent the standard error of the mean (SEM) from three independent experiments. **C**. Immunostaining shows increased Vimentin and N-cadherin expression in the cytoskeleton during TGFβ-induced EMT. **D**. Immunostaining shows the cells with Vimentin (Green) expression have less or no Runx1 (Red) expression.

MCF10A cells were incubated with 10 ng/ml TGFβ1 for 6 days, and we observed that the original cobblestone-like epithelial morphology with tight cell-cell contact was lost, and cells gained an elongated fibroblast-like morphology (Figure 2A). When the levels of epithelial and mesenchymal markers were examined by western blotting and immunofluorescence microscopy, the TGFβ1 treated cells exhibited a 50% down-regulation of the epithelial marker E-cadherin, while expression of the mesenchymal markers Vimentin and N-cadherin was induced (Figure 2B). Significantly, in this TGFβ induced EMT model, we observed the down regulation of Runx1 in both protein and mRNA levels (Figure 2B). Although the immunofluorescence results showed that not all cells acquired the mesenchymal phenotype (Figure 2C), indicating that only a subset of the cells underwent EMT, we still find that Runx1 is decreased during EMT. As further evidence that loss of Runx1 occurs concomitantly with EMT, co-immunofluorescence reveals that the subset of cells undergoing EMT (Vimentin positive cells), had lower or no Runx1 expression (Figure 2D). These results support the idea that Runx1 may function as a suppressor for the EMT.

### Runx1 rescues the TGFβ-induced EMT phenotype

To further prove a functional role for Runx1 in preventing EMT and maintaining the epithelial phenotype, we examined whether overexpressing Runx1 could reverse the EMT phenotype after TGFβ induction.

**Figure 3:**
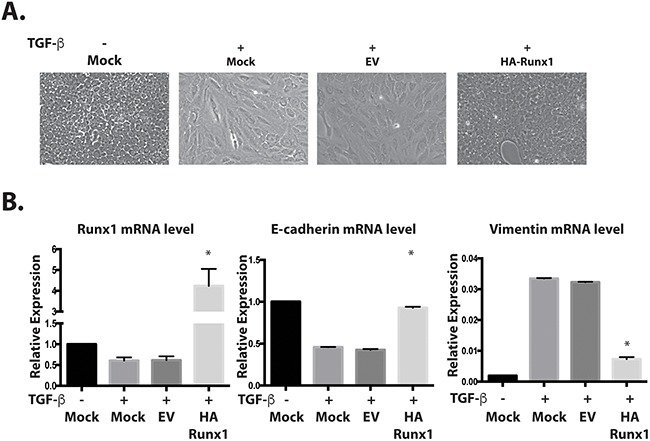
Runx1 reverses TGFβ induced EMT **A**. Images of MCF10A cells treated with TGFβ show morphological changes toward an EMT-like state. Overexpressing Runx1 in TGFβ treated cells rescued cell morphology to an epithelial-like state. **B**. RT-qPCR of RNA from MCF10A cells show changes in gene expression by overexpressing Runx1 in TGFβ-treated cells, which activates E-cadherin and represses Vimentin expression. Student's *t* test * *p* value <0.05 for HA-Runx1 overexpression in MFC10A cells compared to EV control cells. Error bars represent the standard error of the mean (SEM) from three independent experiments.

A plasmid containing HA-tagged Runx1 was transfected into TGFβ treated MCF10A cells. We observed that the cells with Runx1 overexpression changed their morphology from mesenchymal-like back to epithelial-like (Figure 3A). Overexpressing Runx1 in these cells also increased E-cadherin and repressed Vimentin expression, suggesting that cells re-acquired an epithelial phenotype and that the TGFβ induced EMT was blocked (Figure 3B). This result demonstrated that the repression of Runx1 is a necessary step during TGFβ induced EMT.

### Decreased expression of Runx1 during TGFβ independent EMT in MCF10A cells

**Figure 4:**
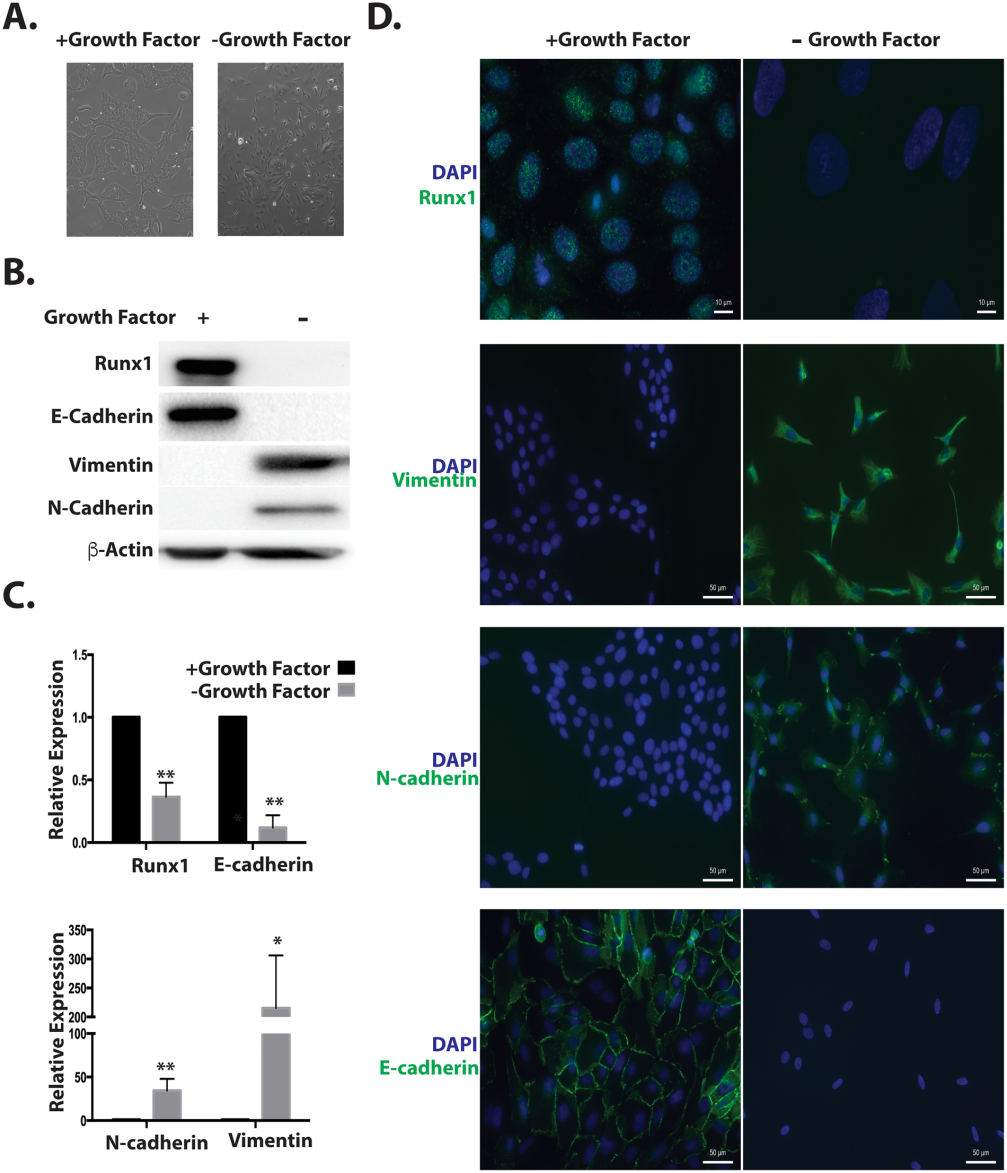
Decreased Runx1 during TGFβ-independent EMT **A**. Images of MCF10A cells grown in medium without growth factors (Insulin, EGF, Hydrocortisone and Cholera toxin) for 7 days show morphological changes from cobblestone to spindle-like. **B**. Western blot analyses of cell lysates from MCF10A cells treated with or without growth factors show changes in EMT markers and Runx1 expression during EMT. **C**. RNA expression of the EMT markers E-cadherin, N-cadherin and Fibronectin was quantified using RT-qPCR in MCF10A cells in the presence or absence of growth factors. Student's *t* test * *p* value <0.05, ** p value <0.01 for growth factors depleted MCF10A cells compared to cells with growth factors. Error bars represent the standard error of the mean (SEM) from three independent experiments. **D**. Immunostaining of E-cadherin, Vimentin, N-Cadherin and Runx1 reveals changes in organization of cell–cell adhesion, cytoskeleton and decreased Runx1.

We considered the possibility that Runx1 may function in a TGFβ independent manner to repress EMT. We used a cell model that is independent of exogenous TGFβ. It has been previously shown that withdrawal from MCF10A medium of specific factors required for optimal cell growth (insulin, EGF, Hydrocortisone and Cholera Toxin), changed cell morphology from cobblestone to spindle like [20]. Here we demonstrate that this morphological change (Figure 4A) resembles an EMT process. Western blotting and qRT-PCR results show that the epithelial marker E-cadherin was down regulated, while mesenchymal markers N-cadherin and Vimentin were upregulated (Figure 4B and 4C). Importantly Runx1 protein is not detected in growth factor depleted cells by western blot and immunofluorescence microscopy (Figure 4B and 4D, top panel). Compared with TGFβ induced EMT (Figure 2C), in this TGFB independent model, all cells acquired the mesenchymal phenotype and lost epithelial mark and Runx1 expression (Figure 4D). These results reveal that modifying growth medium is a more powerful method for inducing EMT in MCF10A cells. Based on the loss of Runx1 during both TGFβ-dependent and independent EMT, we conclude that Runx1 is a key factor in repressing the EMT and maintaining epithelial morphology in normal-like mammary epithelial cells.

### Gene expression profiling of growth factor-depleted MCF10A cells reveals the spectrum of EMT markers

**Figure 5:**
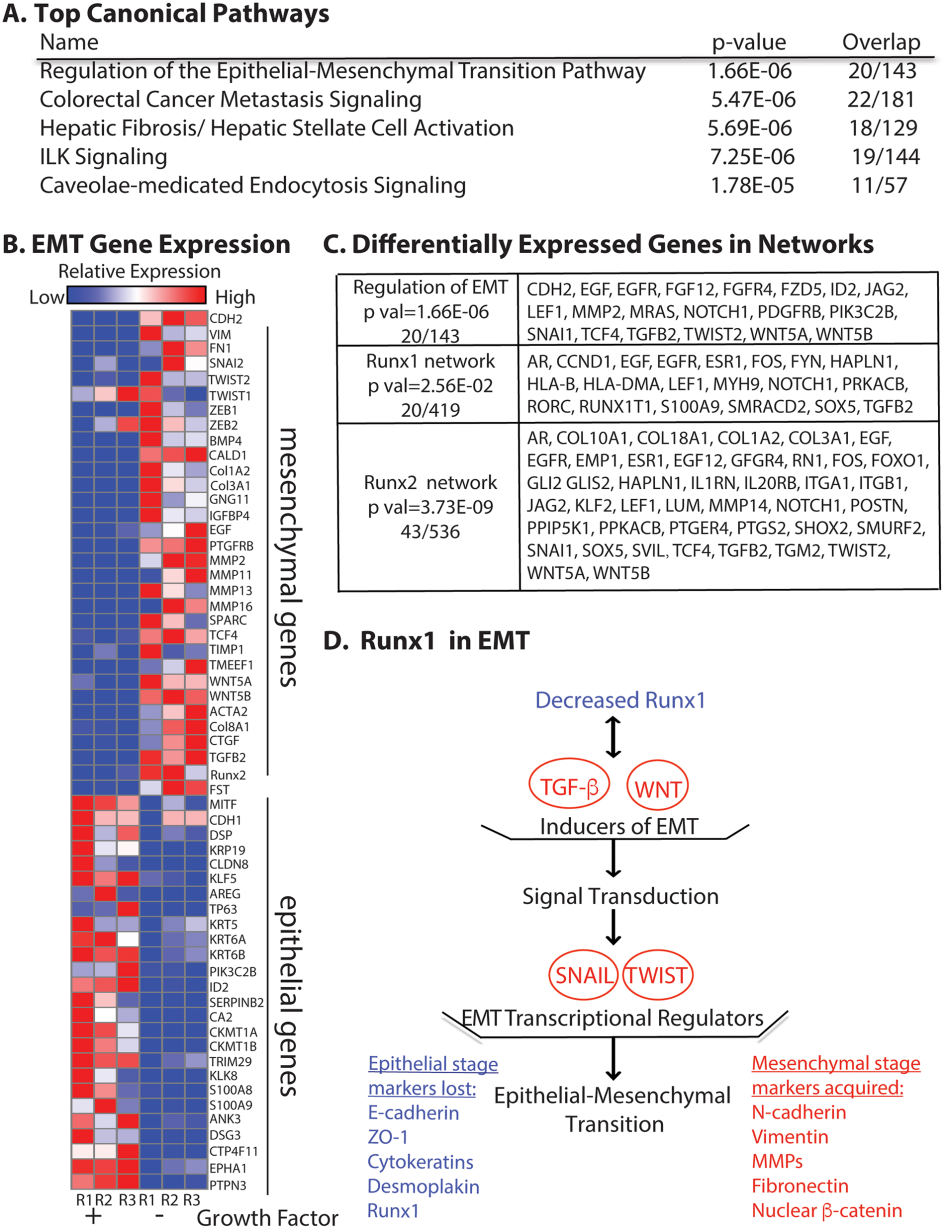
RNA-Seq reveals MCF10A cells undergo EMT upon growth factor removal **A**. Top canonical pathways with the most significant p values identified by using Ingenuity Pathway Analysis (QIAGEN, Hilden, Germany). **B**. Relative expression heat map of 58 EMT related genes confirming MCF10A cells undergo EMT. **C**. Differentially expressed genes (2-fold cut off) in the EMT regulation pathway (p val 1.66E-06), Runx1 interaction network (p val 2.56E-02) and Runx2 interaction network (p val 3.73E-09). **D**. Model of Runx1 function in growth factor depletion induced EMT. Illustration shows the consequences of up and down regulated genes when Runx1 is decreased upon growth factor depletion. The listed genes and pathways are promoting EMT by loss of Runx1 function. Blue indicates down regulated genes. Red indicates up regulated genes or pathways. Ingenuity Pathway Analysis (QIAGEN) was used in panel **A**, **C** and **D**; GENE-E (Broad Institute, Cambridge, MA, USA) was used in panel **B**.

To further understand the mechanisms of growth factor depletion induced EMT, we carried out unbiased genome-wide expression profiling by RNA-Seq, comparing cells grown in normal and growth factor depleted conditions. Among the 1880 differentially expressed mRNAs that have a 2-fold cut off, 457 genes were up- and 1423 were down-regulated. Gene ontology analysis identified functional categories and associated pathways (Figure 5). Among the top 5 canonical pathways that were affected, regulation of the EMT pathway was the most significant with 20 genes altered in the network (Figure 5A and 5C). This observation further confirmed that this novel method of removing growth factors in MCF10A induces EMT. Other relevant pathways include cancer metastasis signaling and integrin-like kinase (ILK) signaling (Figure 5A). Together these most significant signaling pathways are indicative of the MCF10A cells acquiring a more cancer related phenotype.

In addition to pathway analysis, we selected 58 epithelial and mesenchymal genes by using two database sources (described in Materials and Methods) and examined the expression patterns based on relative reads from our RNA-Seq profiling. The heat map constructed from these data (Figure 5B) compares expression of EMT genes under two different growth conditions—normal and growth factor-depleted. Well-established epithelial genes such as DSP, Claudins and KRT family [21] were down regulated. We observed consistent up-regulation of common mesenchymal genes (CDH2, FN1 and VIM) as well as genes related to signaling pathways such as BMP/TGFB and WNT when growth factors were removed. We also noted that both TGFβ2 and Runx2 are among up-regulated genes (Figure 5B). Moreover, we found that expression of 43 genes in the Runx2 interaction network were altered (Figure 5C), consistent with up-regulation of Runx2 protein level upon growth factor depletion (Supplementary Figure 1) and its role in promoting invasion and metastasis to bone [5].

To study how loss of Runx1 is involved in this EMT process, we also examined the Runx1 interaction network and found that 20 genes (Figure 5C) were altered upon growth factor depletion. Further pathway analysis with the 1880 differentially expressed genes revealed that decreased Runx1 and the altered Runx1 interaction network are associated with activation of TGFβ and WNT pathways (Figure 5D), which are known to relate to Runx1 function [22]. The stimulated TGFβ and WNT pathways further activate the downstream well-studied EMT-inducing transcription factors Snail and Twist (Figure 5D) [21]. These studies provide evidence that depletion of Runx1 contributes to initiation of EMT in the normal-like MCF10A mammary epithelial cells. These results also indicate that Runx2 plays an important role during growth factor-starvation induced EMT and elucidate mechanisms by which Runx1 and Runx2 are involved in EMT. Together, these RNA-Seq data confirm that the growth factor-starvation method is a unique cell treatment to induce EMT in MCF10A cells without exogenous addition of TGFβ.

### Directly depleting Runx1 in MCF10A cells results in loss of epithelial morphology and activation of EMT

**Figure 6:**
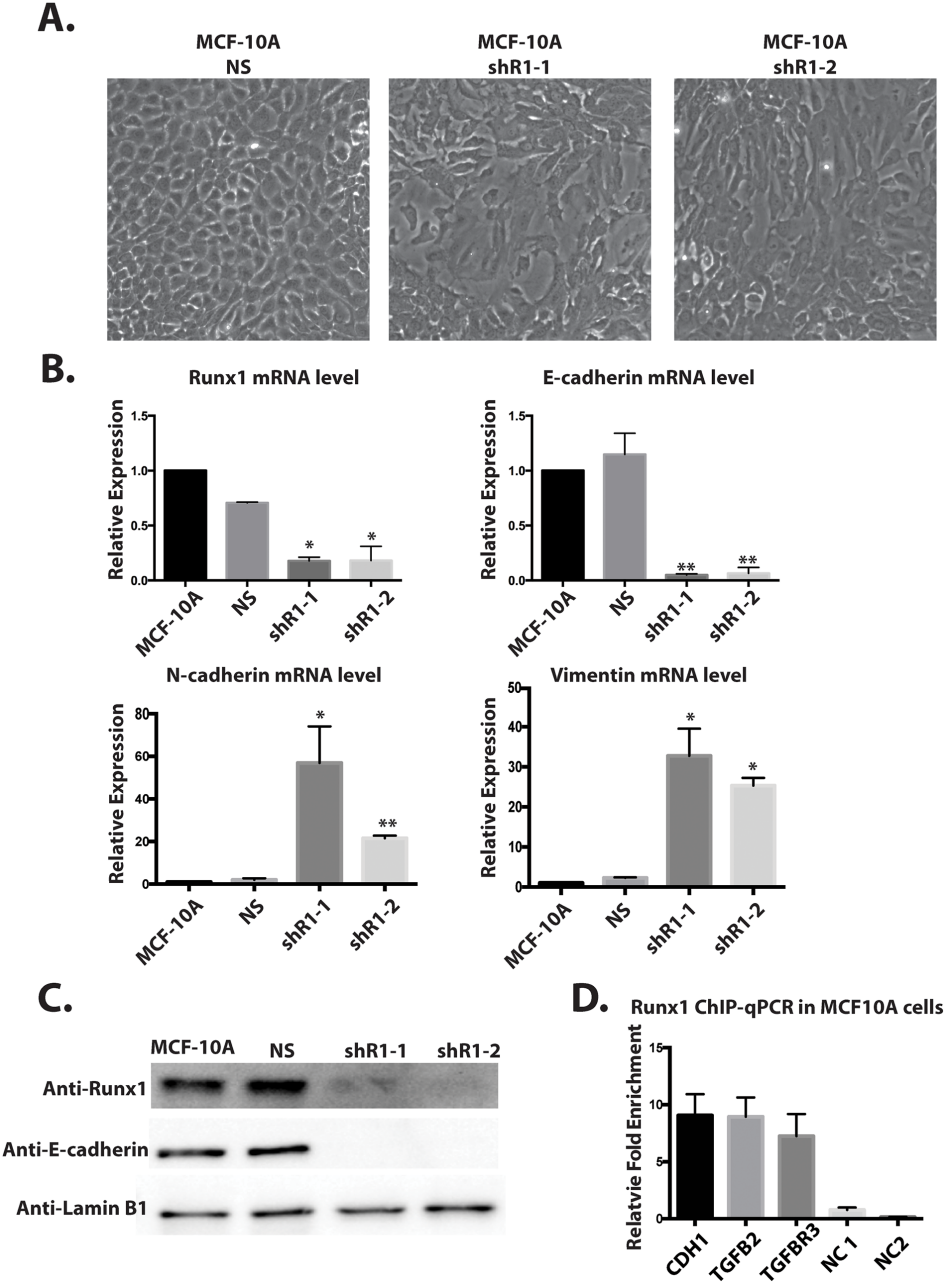
Depleting Runx1 in MCF10A cells promotes a mesenchymal-like phenotype **A**. MCF10A cells treated with shRunx1 show morphological changes toward an EMT- like state. **B**. Western blot analyses of lysates from MCF10A cells treated with shRunx1 show decreased protein expression of Runx1 and E-cadherin. **C**. RT-qPCR analyses of RNA from MCF10A cells treated with shRunx1 show decreased gene expression of E-cadherin and activation of mesenchymal marks of N-cadherin and Vimentin. Student's *t* test * *p* value <0.05, ** p value <0.01 for MCF10A shRunx1 cells compared to the MCF10A ns cells. Error bars represent the standard error of the mean (SEM) from three independent experiments. **D**. ChIP-qPCR confirmation of Runx1 occupancy at CDH1, TGFB2 and TGFBR1. ZNF188 (NC1) and ZNF333 (NC2) were used as the negative control as Runx1 are predicted not to bind these genes. Data obtained with antibodies against Runx1 are normalized to input control.

We have shown by multiple lines of evidence that down-regulation of Runx1 is a key step during breast cancer EMT. However, we still could not distinguish whether decreased Runx1 expression drives the activation of EMT or is an outcome of EMT. To address that question and understand whether Runx1 can function directly to maintain normal epithelial morphology, we inhibited endogenous Runx1 expression in MCF10A cells using lentivirus that contained short-hairpin RNA targeting Runx1 (shRunx1) (Figure 6). We generated two different MCF10A shRunx1 cell lines using two different shRNA sequences (shR1-1, shR1-2). Compared to the parental and control (non-silencing) cells, we observed that Runx1-depleted MCF10A cells showed an obvious shift in morphology from cobblestone-like cells to more spindle-shaped cells (Figure 6A). Western blot and Q-PCR analysis demonstrated endogenous Runx1 was down regulated at both the protein and mRNA levels (Figure 6B and 6C). Because the shRunx1 cells exhibited a morphological change consistent with loss of the epithelial phenotype, E-cadherin expression was examined. Runx1 knockdown cells showed a significant decrease of E-cadherin, as well as up-regulation of the mesenchymal genes Vimentin and N-cadherin (Figure 6C).

Taken together, these results indicate that depletion of Runx1 directly initiates EMT in MCF10A cells, and establishes for the first time that Runx1 is required to maintain the normal mammary epithelial phenotype. The mechanism for these biological activities involves Runx1 binding to EMT-related target genes.

Previously it has been shown that both E-cadherin [23] and genes in TGFB family [24] have Runx1 binding sites. Thus to further support a direct role for Runx1 regulation of E-cadherin and TGFβ signaling in MCF10A cells, a Runx1 ChIP-qPCR was performed (Figure 6D). Significant enrichment of Runx1 binding on E-cadherin (CDH1), TGFB2 and TGFBR3 genes were observed. The positions of the amplicons on tested genes are shown in Supplementary Figure 2. These results indicate that Runx1 may directly bind to the E-cadherin gene and regulate its expression. Our findings also provide an additional line of evidence for a key function of Runx1 in blocking TGFβ signaling and maintaining epithelial morphology. Further the binding of Runx1 to the E-cadherin gene is also associated with the H3K4ac activating histone mark [25]. We searched for putative Runx1 binding sites and found 5 consensus motif sequences which are coincident with H3K4ac peaks present in MCF10A cells, but not in metastatic MDA-MB-231 cells (Supplementary Figure 3).

### Depleting Runx1 in MCF7 breast cancer cells promotes EMT

**Figure 7:**
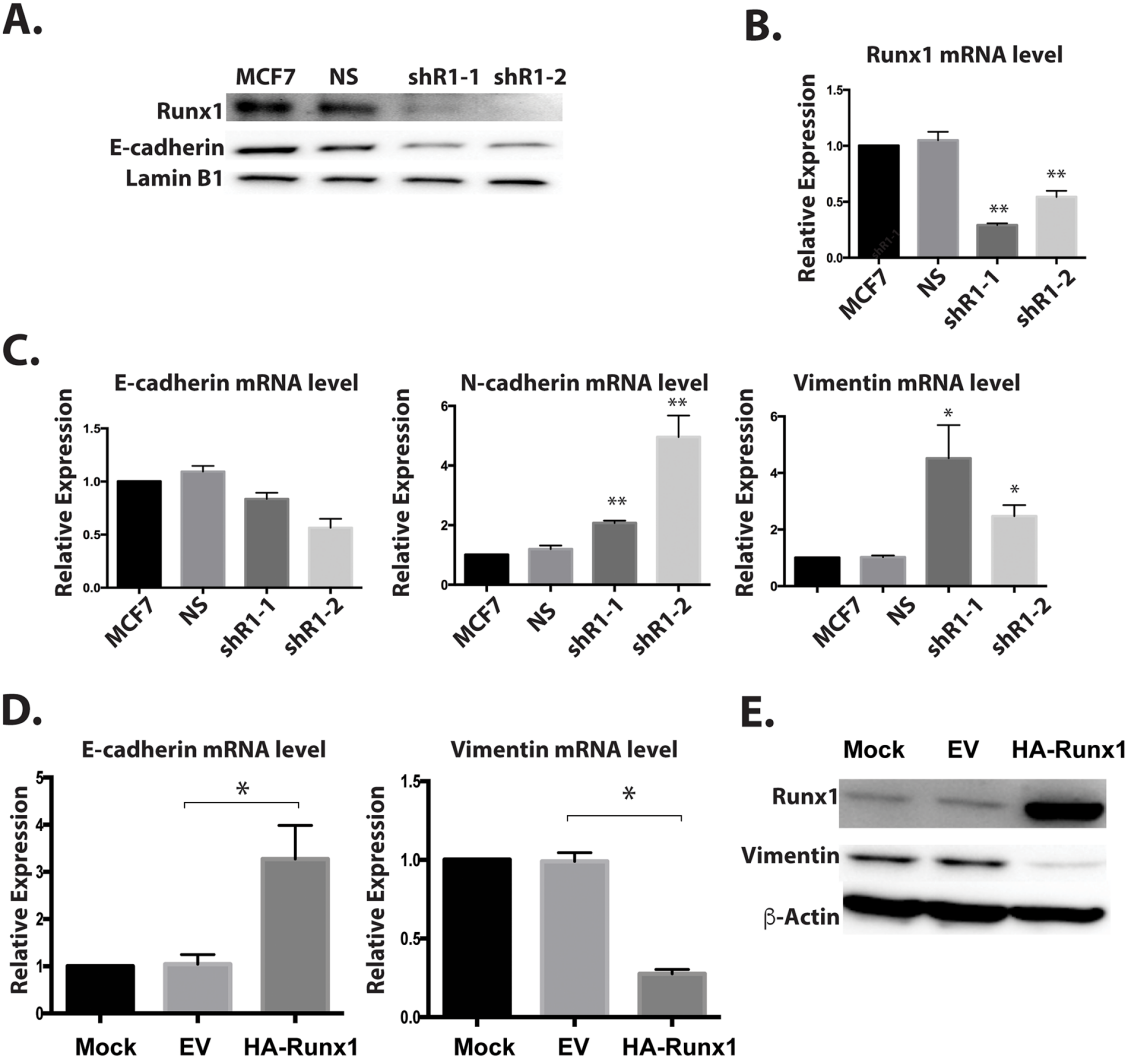
Runx1 controls EMT-MET in non-metastatic breast cancer cells Two breast cancer cell lines MCF7 (epithelial-like) **A**-**C**. and MCF10AT1 (mesenchymal-like) **D**, **E**. were examined for Runx1 knockdown or ectopic expression, respectively. **(A)** Western blot analyses of lysates from MCF7 cells with Runx1 depletion show decreased protein expression of Runx1 and E-cadherin. **(B)** RT-qPCR of RNA from MCF7 cells treated with shRunx1 shows decreased gene expression of Runx1. **(C)** RT-qPCR shows decreased gene expression of E-cadherin and increased gene expression of N-cadherin and Vimentin in Runx1 depleted MCF7 cells. Student's *t* test * *p* value <0.05, ** p value <0.01 for MCF7 shRunx1 cells compared to the MCF7ns cells. Error bars represent the standard error of the mean (SEM) from three independent experiments. **(D)** RT-qPCR of RNA from MCF10AT1 cells overexpressing Runx1 show increased gene expression of E-cadherin and decreased gene expression of Vimentin. Student's *t* test * *p* value <0.05 for MCF10AT1 Runx1 overexpression cells compared to the MCF10AT1 EV cells. Error bars represent the standard error of the mean (SEM) from three independent experiments. **(E)** Western blot analyses of lysates from MCF10AT1 cells treated with Runx1 overexpression show increased protein expression of Runx1 and decreased expression of Vimentin.

The loss of epithelial morphology in normal-like mammary cells by knockdown of Runx1 (Figure 6) raises a compelling question regarding the role of Runx1 in breast cancer cells. Therefore, we tested whether this regulation also occurs in epithelial-like MCF7 breast cancer cells. Two shRunx1 (shR1-1, shR1-2) stable knockdowns in the MCF7 cell line were generated. Endogenous Runx1 was down regulated at both the protein and mRNA levels for both short-hairpin RNAs (Figure 7A and 7B). In these Runx1-depleted MCF7 cells, western blot and RT-Q-PCR analyses revealed a significant decrease of E-cadherin expression at both the protein and mRNA levels and up-regulation of the mesenchymal genes Vimentin and N-cadherin at the mRNA level (Figure 7C). Based on these results, we conclude that Runx1 is preventing EMT in both normal mammary cells (MCF10A) and breast cancer cells (MCF7), consistent with its function in maintaining an epithelial phenotype.

### Overexpressing Runx1 in mesenchymal like breast cancer cells drives mesenchymal to epithelial transition (MET)

To further establish a definitive role for Runx1 function in preserving the epithelial phenotype, we carried out a “rescue” study to examine the consequences of restoring Runx1 expression in mesenchymal like breast cancer cells (Figure 7D and 7E). Runx1 was ectopically expressed in tumorigenic MCF10AT1 cells, which resulted in increased E-cadherin expression and decreased Vimentin expression (Figure 7D and 7E). Notably, the E-cadherin level is only increased at mRNA level but not protein level under transient transfection conditions (data not shown). This key finding shows that overexpression of Runx1 in mesenchymal cancer cells drives the cells back to the epithelial stage. These observations provide direct evidence that Runx1 prevents EMT.

### Runx1 expression in breast tumors correlates with metastasis, tumor subtype and survival

**Figure 8:**
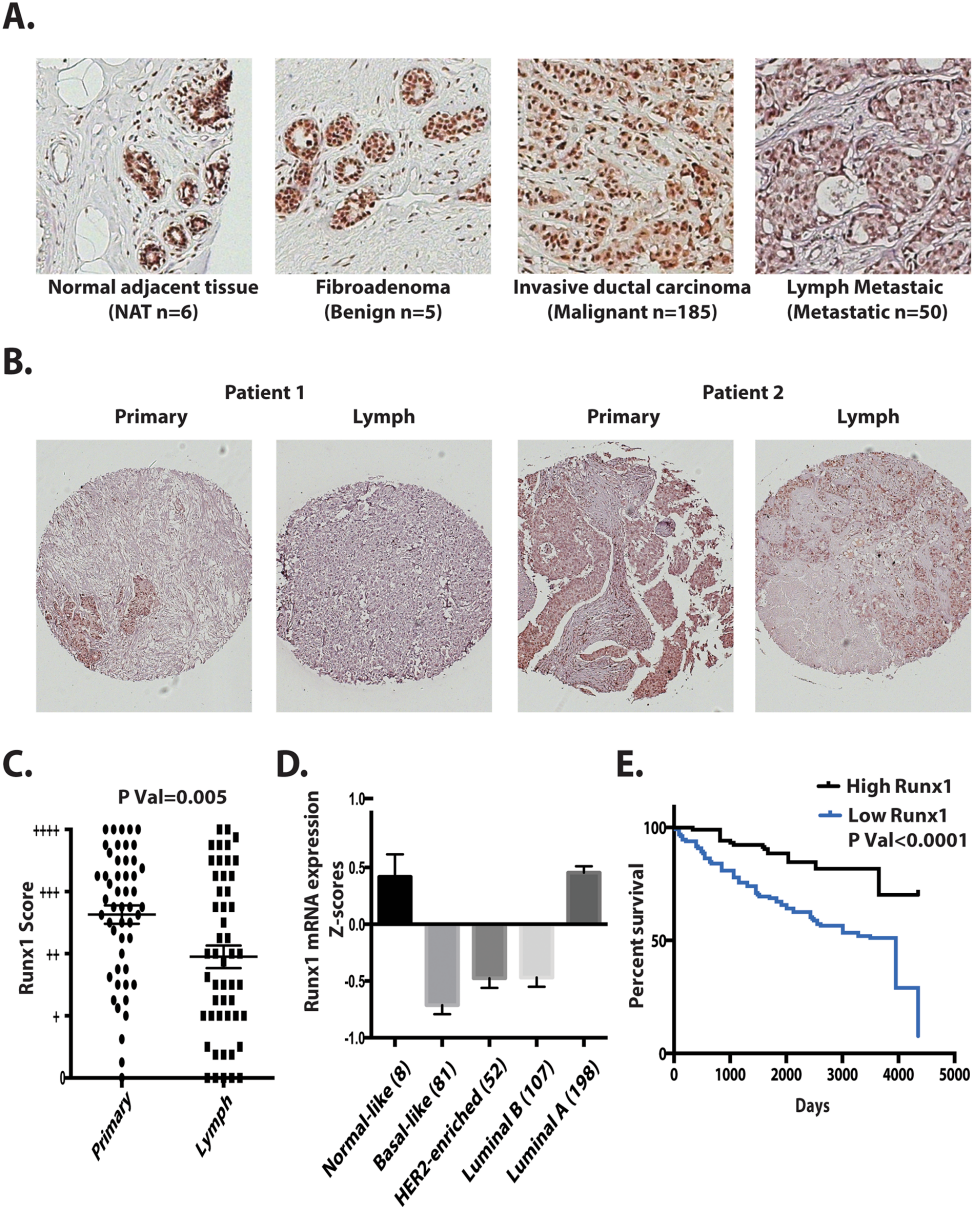
Runx1 expression in breast tumors correlates with metastasis, tumor subtype and survival **A**. Representative tissue microarray images of Runx1 in normal adjacent tissue (NAT), fibroadenoma, invasive ductal carcinoma, and tumor metastasis to lymph. **B**. Representative of TMAs (n=50) showing two patients’ primary tumor and their lymph metastasis with Runx1 positive cells (brown stain). Two tailed t test ** p<0.005 between primary tumor and lymph metastatic sites. **C**. Distribution of Runx1 staining scores for 50 patients with primary breast tumor and lymph metastasis. Using a semi-quantitative scoring system, three researchers blindly scored TMAs. **D**. Runx1 mRNA is decreased in breast cancer subtypes. **E**. Kaplan-Meier analysis showed higher overall survival in patients with higher Runx1 mRNA expression (GSE3494-U133A). Gehan-Breslow-Wilcoxon test with *p* value<0.0001 compared with high Runx1 expression patients and low Runx1 expression patients.

We next evaluated Runx1 expression in breast cancer patient tissues. With a highly specific Runx1 antibody, we applied immunohistochemistry to determine the expression pattern of Runx1 in different types of breast cancer using a Tissue Microarray (TMA) of 185 tumors and 6 control normal adjacent tissue sections. The results identified that Runx1 expression is associated with breast cancer stages and subtypes. We observed Runx1 expression at high levels in all normal and benign mammary epithelial tissues (Figure 8A). Runx1 is also expressed in breast cancer samples including ductal carcinoma *in situ* and invasive ductal carcinoma (Figure 8A). However, breast cancer cells metastatic to the lymph node showed significantly less Runx1 expression compared with the primary tumor site (Figure 8A and 8B). Quantification of Runx1 levels at primary sites and lymph metastatic sites in 50 patients showed that Runx1 is significantly lower (p=0.005 using two tailed t test) in lymph samples (Figure 8C). We also observed slightly higher Runx1 levels in grade 1 compared with grade 2 tumors (Supplementary Figure 4).

We further investigated the relationship of Runx1 expression to clinical outcomes through mining of The Cancer Genome Atlas (TCGA) database. Runx1 was found to be under-expressed in several breast cancer subtypes, including Luminal B, Her2 enriched and basal like breast cancers, which all have a poor prognosis (Figure 8D). Luminal A subtype, which is generally associated with a good prognosis, showed Runx1 levels equivalent to normal-like breast tissue. However 5% of samples in this subtype have Runx1 somatic mutations [10], with the majority located in the Runx1 DNA-binding domain, which can compromise Runx1 transcriptional activity. We conclude from these data that Runx1 expression is subtype-dependent and correlates with prognosis.

Runx1 expression levels were also compared with patient survival rates using a data set (GSE3494-U133A) in the Gene Expression Omnibus database (Figure 8E). Our analyses shows that patients with low Runx1 levels in their tumors exhibit poor survival relative to patients with high Runx1 expression.

Taken together our data demonstrate that Runx1 functions as a tumor suppressor in normal epithelial cells, by sustaining the epithelial phenotype and preserving the epithelial integrity. Loss of Runx1 is not only accompanied with EMT (Figures 2-5) but can also initiate the transformation process (Figures 6 and 7). Therefore, loss of Runx1 normal activities in tumor tissues may serve as an indicator of poor prognosis for breast cancer patients as revealed in several clinical studies (Figure 8). We conclude from these clinical data that as tumors advance from early stage to a more aggressive phenotype, loss of Runx1 may promote tumor progression.

## DISCUSSION

Our study has established a crucial role for Runx1 in maintaining the normal epithelial phenotype. This finding is supported by our demonstration that Runx1 is decreased during EMT and that loss of endogenous Runx1 initiates and promotes EMT which is also accompanied by changes in the morphology of mammary epithelial cells. Using two independent methods to induce EMT, either by adding TGFβ or removing required growth factors which increases/activates TGFβ expression, we observed significantly decreased Runx1 expression. Further, Runx1 re-expression rescues the epithelial phenotype following TGFβ treatment, which assures maintenance of normal epithelial cell morphology and prevents EMT. By inhibition of Runx1 in MCF10A (normal) and MCF7 (epithelial like breast cancer) cells, together with re-expression in MCF10AT1 (malignant cells with low Runx1 levels), we provide direct evidence that loss of Runx1 directly contributes to the initiation of EMT in breast cancer, while the presence of Runx1 restores the epithelial phenotype. Together these findings have revealed, for the first time, that the expression of Runx1 has a critical function in preserving epithelial morphology in mammary epithelial cells and preventing EMT; thus, Runx1 can be considered as a tumor suppressor in normal epithelial cells.

Here we focused our study on normal mammary epithelial and epithelial like breast cancer cells, and discovered a key function for Runx1 in preventing EMT. We examined the mechanisms by which Runx1 regulates EMT in cancer progression. First, we show Runx1 is a positive regulator of the epithelial marker E-cadherin. Upon loss of Runx1, the expression level of E-cadherin is strikingly decreased. We also showed that Runx1 directly binds to a consensus motif in the E-cadherin gene using ChIP-qPCR. Second, we demonstrate Runx1 operates downstream of the TGFβ pathway and functions as a suppressor of TGFβ regulation. Runx1 is well established to mediate TGFβ-BMP signaling by forming co-regulatory complexes with SMADs [19, 26]. Our RNA-Seq analysis of growth factor-depleted cells suggests that loss of Runx1 is coupled with activation of the TGFβ pathway. This was confirmed experimentally by showing that Runx1 is decreased upon TGFβ treatment and Runx1 rescues TGFβ induced EMT. Supporting these molecular mechanisms, Runx1 has known properties that establish cell phenotypes, including the hematopoietic lineage [27], regulating quiescent hair follicle bulge stem cells to differentiate to early progenitor hair germ cells [28]. Very recently Runx1 was shown to be transiently upregulated early in hESC differentiation to mesendodermal lineages via Runx1-TGFB2 signaling and that loss of Runx1 impaired epithelial differentiation [29]. Thus our studies, which have now identified a cellular function for Runx1 in normal mammary cells, are consistent with these other normal tissues to support their cell type specific phenotype. We have further studied the consequence of disturbing normal Runx1 function in breast cancer cells and provided evidence that Runx1 loss of function has a significant effect on cancer-related mechanisms.

Repression, overexpression, and/or deregulated functioning of Runx1 have been shown to cause cancers [30]. TGFβ is a well-known EMT inducer and has a dual role in breast cancer progression [31]. In normal epithelial cells and early stage breast cancer, TGFβ acts as a tumor suppressor, yet at later stages of tumor progression can promote cancer cell migration, invasion and metastasis [32]. Our results have provided evidence that TGFβ is an upstream regulator of Runx1. Because Runx1 is downstream of TGFβ, Runx1 may also have different functions depending on the specific cellular context [33]. For example, while Runx1 has been shown to function as a tumor suppressor in prostate cancer [34], it acts as an oncogene in ovarian cancer [35] and in a mouse model of breast cancer [33]. Our identification of TGFβ as a Runx1 upstream regulator provides insight into the compromised mechanisms of Runx1 function that are associated with breast cancer.

Runx1 is also subject to the hormonal status of cells. Treating ER+ breast cancer cells with 17β-estradiol promotes EMT [36] and also decreases Runx1 expression [37]. In turn, depletion of Runx1 represses the expression of estrogen receptor α [38], suggesting a negative feedback loop in progression of ER+ breast cancer. Our data show MCF7 ER+ breast cancer cells can be induced into EMT by Runx1 depletion. One study using computational analysis revealed that Runx1 is highly correlated with mammary stem cell differentiation [39]. Other studies showed that Runx1 is important for mammary gland maturation, and its interaction with ERα is necessary for luminal development and may prevent breast cancer progression [38, 39]. It also has been shown that Runx1 represses WNT pathways, which allows ER to be expressed in luminal breast cancer cells [22]. All these pieces of evidence raise the hypothesis that Runx1 could function as a tumor suppressor in ER positive breast cancer; here we clearly demonstrate Runx1 has a direct role to prevent EMT in MCF7 ER+ breast cancer cells, and thus establishes Runx1 as a tumor suppressor.

In addition to Runx1-mediated mechanisms downstream of TGFβ (feedback loop) and upstream hormonal regulation of Runx1, miRNAs are also a likely mechanism contributing to the down regulation of Runx1 during EMT. MicroRNAs are known to promote/inhibit EMT (e.g., miR-200 family, miR-27 and miR-30) [40]. Our analysis using TargetScan7.0 indicates that most of these miRNAs also target the Runx1 3’UTR. It has been shown that miR27a [41], miR144 [37] and miR387 [42], which are upregulated during breast cancer progression, are directly down-regulating Runx1. The convergence of these multiple pathways that inhibit Runx1 expression leads us to conclude that loss of Runx1 is an important mechanistic step in breast cancer initiation and/or progression.

Examination of TCGA and other public datasets identified loss of Runx1 correlates with poor prognosis (Figure 8C) and poor survival (Figure 8D). It has been shown in breast tumors that the majority of EMT markers are expressed in basal layer cells [43]. Also reported is that basal subtypes of breast cancer are more aggressive and metastatic compared to the luminal subtypes [44]. TCGA data show that Runx1 is expressed at the lowest level in patients with basal like breast cancer. These findings are consistent with our identification of a Runx1 function in preserving the epithelial phenotype in normal like basal cells (MCF10A). Loss of Runx1 expression may cause the basal cells to lose their epithelial morphology, phenotype integrity and become more susceptible to initiation of EMT. Therefore, our functional studies focused on the role of Runx1 in basal-like mammary epithelial cells (MCF10A).

Intact Runx1 function is also important for Luminal A breast cancer. Genetic studies show Runx1 is mutated in 5% of Luminal A subtype breast cancer patients [10, 11]. A recent study suggested that in MCF7 cells, disruption of Runx1 function might contribute to development of ER^+^ luminal breast cancer in the context of either *TP53* or *RB1* loss [38]. Significantly, we demonstrated that loss of Runx1 in luminal like breast cancer cells (MCF7) can promote EMT (Figure 7). Taken together, these biochemical and clinical data support the emerging concept that Runx1 is a tumor suppressor and that loss of Runx1 is associated with the progression of breast cancer.

Our studies demonstrate a clear reduction of endogenous Runx1 in two cell models (MCF7 and MCF10AT1) of breast cancer. This finding is consistent with human TMA data that show the strongest Runx1 staining (66% strong or moderate levels) in normal cases, compared with 29% and 35% in DCIS and IDC samples, respectively [43, 44]. However, this human data is in contrast to findings in the MMTV-PyMT mouse model of breast cancer [33], where Browne et al. reported that Runx1 steadily increased during tumor growth. Thus, the decreased Runx1 in human samples with increased disease progression indicates Runx1 has distinct functional activities that differ between mouse and human breast tumors.

In conclusion, we identified Runx1 as a key transcription factor in basal epithelial breast cells through its ability to maintain normal epithelial morphology. Our studies offer Runx1 as a novel bio-therapeutic molecule for breast cancer intervention.

## MATERIALS AND METHODS

### Cell lines and cultures

Human breast cancer cell lines MCF10A, MCF7, MDA-MB-231 and T47D cells were purchased from ATCC. MCF10AT1 and MCF10CA1a cells are a gift from Jeff Nickerson's lab.

MCF10A cells were grown in DMEM: F12 (Hyclone: SH30271, Thermo Fisher Scientific, Waltham, MA, USA) with 5% (v/v) horse serum (Gibco: 16050, Thermo Fisher Scientific, Waltham, MA, USA) + 10 μg/ml human insulin (Sigma Aldrich, St. Louis, MO: I-1882) + 20 ng/ml recombinant hEGF (Peprotech, Rocky Hill, NJ, USA: AF-100-15) + 100 ng/ml cholera toxin (Sigma Aldrich: C-8052) + 0.5 μg/ml hydrocortisone (Sigma Aldrich: H-0888) 50 IU/ml penicillin/50 μg/ml streptomycin and 2 mM glutamine (Life Technologies, Carlsbad, CA, USA: 15140-122 and 25030-081, respectively). TGFβ induced EMT in MCF10A cells was initiated by addition of 10 ng/ml TGFβ1 (R&D Systems, Minneapolis, MN, USA) to the medium. Growth factors starvation induced EMT in MCF10A cells was performed as previously described [16]. Briefly, MCF10A cells were plated in completed medial and at day 2, the medium was switched to DMEM: F12, with 5% (v/v) horse serum and 50 IU/ml penicillin/50 μg/ml streptomycin without added growth factors. The cells were maintained in this medium for up to 14 days until the morphological change was observed.

MCF10AT1 cells were grown in the same medium as MCF10A cells. MCF10CA1a cells were grown in DMEM: F with 12, 5% (v/v) horse serum with 50 IU/ml penicillin/50 μg/ml streptomycin and 2 mM glutamine. MCF7 cells were maintained in Dulbecco modified Eagle medium (DMEM) high glucose (Fisher Scientific: Thermo Fisher Scientific, Waltham, MA, USA: MT-10-017-CM) supplemented with 10% (v/v) FBS (Atlanta Biologicals, Flowery Branch, GA, USA: S11550), 50 IU/ml penicillin/50 μg/ml streptomycin. T47D cells were maintained in RPMI 1640 with phenol red (Fisher Scientific: MT-10-040-CM) supplemented with 10% (v/v) FBS and 50 IU/ml penicillin/50 μg/ml streptomycin. MDA-MB-231 cells were cultured in alpha minimal essential medium (α-MEM) (Life Technologies: A10490-01) containing 10% (v/v) FBS and 50 IU/ml penicillin/50 μg/ml streptomycin. MCF10CA1a cells were transfected using FuGENE-6 (Roche, Indianapolis, IN, USA) according to the instructions of the manufacturer.

### Lentiviral plasmid preparation and viral vector production

Lentivirus-based RNAi transfer plasmids with pGIPZ shRunx1 (clone V2LHS_150257 and V3LHS_367631, GE Dharmacon) and pGIPZ non-silencing (Cat No. RHS4346, GE Dharmacon) were purchased from Thermo Scientific. To generate lentivirus vectors, 293T cells in 10 cm culture dishes were co-transfected with 10 μg of pGIPZ shRunx1 or pGIPZ non-silencing, with 5 μg of psPAX2, and 5 μg of pMD2.G using lipofectamine 2000 reagent (Life Technologies). Viruses were harvested every 48 h post-transfection. After filtration through a 0.45 μm-pore-size filter, viruses were concentrated by using LentiX concentrator (Clontech, Mountain View, CA, USA).

### Gene delivery by transfection and infection

For shRNA-mediated knockdown of Runx1 expression, MCF10A or MCF7 cells were plated in six-well plates (1×10^5^ cells per well) and infected 24 h later with lentivirus expressing shRunx1 or nonspecific shRNA. Briefly, cells were treated with 0.5 ml of lentivirus and 1.5 ml complete fresh DMEM-F12 per well with a final concentration of 4 μg/ml polybrene. Plates were centrifuged upon addition of the virus at 1460 × *g* at 37°C for 30 min. Infection efficiency was monitored by GFP co-expression at 2 days post infection. Cells were selected with 2 μg/ml puromycin (Sigma Aldrich P7255-100MG) for at least two additional days. After removal of the floating cells, the remaining attached cells were passed and analyzed.

### Western blotting

Cells were lysed in RIPA buffer and 2X SDS sample buffer supplemented with cOmplete, EDTA-free protease inhibitors (Roche Diagnostics) and MG132 (EMD Millipore San Diego, CA, USA). Lysates were fractionated in an 8.5% acrylamide gel and subjected to immunoblotting. The gels are transferred to PVDF membranes (EMD Millipore) using a wet transfer apparatus (Bio-Rad Laboratories, Hercules, CA, USA). Membranes were blocked using 5% Blotting Grade Blocker Non-Fat Dry Milk (Bio-Rad Laboratories) and incubated overnight at 4°C with the following primary antibodies: a rabbit polyclonal Runx1 (Cell Signaling Technology, Danvers, MA, USA:#4334, 1:1000); a mouse monoclonal to E-cadherin (Santa Cruz Biotechnology, Inc., Santa Cruz, CA, USA: sc21791, 1:1000); a mouse monoclonal Vimentin (Santa-Cruz Biotechnology sc-6260, 1:1000); a mouse monoclonal to β-Actin (Cell Signaling Technology #3700, 1:1000); a rabbit polyclonal LaminB1 (Abcam, Cambridge, UK: 16048, 1:2000); a rabbit polyclonal N-cadherin (Santa Cruz Biotechnology sc-7939, 1:2000). Secondary antibodies conjugated to HRP (Santa Cruz Biotechnology) were used for immunodetection, along with the Clarity Western ECL Substrate (Bio-Rad Laboratories) on a Chemidoc XRS+ imaging system (Bio-Rad Laboratories).

### Immunofluorescence staining microscopy

Cells grown on coverslips were fixed with using 3.7% formaldehyde in phosphate buffered saline (PBS) for 10 min. Cells were then permeabilized in 0.1% Triton X-100 in PBS, and washed in 0.5% Bovine Serum Albumin in PBS. Detection was performed using a rabbit polyclonal Runx1 antibody (Cell Signaling Technology #4336), a mouse monoclonal Vimentin (Santa Cruz Biotechnology sc-6260), a rabbit polyclonal N-cadherin (Santa Cruz Biotechnology sc-7939) and a mouse monoclonal to E-cadherin (Santa Cruz Biotechnology, Inc., Santa Cruz, CA, USA). Staining was performed using fluorescent secondary antibodies; for rabbit polyclonal antibodies a goat anti-rabbit IgG (H+L) secondary antibody, Alexa Fluor® 488 conjugate (Life Technologies A-11008), was used and for mouse monoclonal a F(ab')2-goat anti-mouse IgG (H+L) secondary antibody, Alexa Fluor® 488 conjugate was used (Life Technologies A-11001).

### Quantitative PCR

RNA was isolated with Trizol (Life Technologies) and cleaned by DNase digestion (Zymo Research, Irvine, CA, USA). RNA was reversed transcribed using SuperScript II and random hexamers (Life Technologies). cDNA was then subjected to quantitative PCR using SYBR Green technology (Applied Biosystems, Foster City, CA, USA). Sequences of primers used in the paper. Runx1 Forward: AACCCTCAGCCTCAGAGTCA, Runx1 Reverse: CAATGGATCCCAGGTATTGG; E-cadherin Forward: GGAAGTCAGTTCAGAGCATC, E-cadherin Reverse: AGGCCTTTTGACTGTAATCACACC; N-cadherin Forward: TGTTTGACTATGAAGGCAGTGG, N-cadherin Reverse: TCAGTCATCACCTCCACCAT; Vimentin Forward: AGGAAATGGCTCGTCACCTTCGTGAATA, Vimentin Reverse: GGAGTGTCGGTTGTTAAGAACTAGAGCT; GAPDH Forward: TGTGGTCATGAGTCCTTCCA, GAPDH Reverse: ATGTTCGTCATGGGTGTGAA; HPRT Forward: TGCTGACCTGCTGGATTACA, HPRT Reverse: TCCCCTGTTGACTGGTCATT; β-Actin Forward: AGCACAGAGCCTCGCCTTT, β-Actin Reverse: CGGCGATATCATCATCCAT.

### Tissue microarray

Formalin-fixed paraffin-embedded (FFPE) human breast cancer samples were obtained from the UMMS tissue bank and FFPE human breast cancer tissue microarrays (TMA) from US BioMax (Rockville, MD, USA). TMAs (BR1503a & BR10010) were obtained from US BioMax. Sample information pertaining to Type, Grade, Stage, TNM, were provided by US BioMax. BR1503a is a primary breast tissue array of 150 samples of 75 patient cases: three cases of adjacent normal breast tissue, three cases of breast fibroadenoma, two cases of breast cystosarcoma phyllodes, seven cases of breast intraductal carcinoma, and 60 cases of breast invasive ductal carcinoma. Duplicate cores per case. BR10010 is a breast carcinoma and matched metastatic carcinoma array of 100 samples of 50 patient cases: 46 cases of invasive ductal carcinoma, one case of micropapillary carcinoma, two cases of invasive lobular carcinoma, and one case of neuroendocrine carcinoma. Duplicate cores per case. RUNX1 staining was done as previously described [45] using RUNX1 Rabbit Polyclonal 4334 from Cell Signaling Technology. Each tissue section was imaged and independent researchers blindly scored the sections based on the metric in Figure 8.

### Analysis of Runx1 expression in various cancers using public data sets

Runx1 expression was analyzed in various breast cancer subtype types using the TCGA database (www.cbioportal.org) [10]. The PROGgene database (www.compbio.iupui.edu/proggene) was used to identify the data sets for survival analysis and re-analyzed the public GEO data sets (www.ncbi.nlm.nih.gov/gds) (GSE3494-U133A).

### RNA-Seq, ontology, and pathway analysis

RNA was isolated using DirectZol RNA mini prep kit (Zymo Research), quantified by Qubit HS RNA assay (Thermo Fisher Scientific) and assayed for RNA integrity by Bioanalyzer (Agilent Technologies, Santa Clara, CA, USA). Total RNA was depleted of ribosomal RNA, reverse transcribed and strand-specific adapters added following manufacturer's protocol (TruSeq Stranded Total RNA Library Prep kit with Ribo-Zero Gold, Illumina, San Diego, CA, USA) with the exception that the final cDNA libraries were amplified using the Real-time Library Amplification Kit (Kapa Biosystems, Wilmington, MA, USA) to prevent over-amplification of libraries. Generated cDNA libraries were assayed for quality then sequenced as single-end 100 bp reads (IlluminaHiSeq1000, UVM Advanced Genome Technologies Core). Sequence files (fastq) were mapped to the most recent assemblies of the human genome (hg38) using TopHat2 [46]. Expression counts were determined by HTSeq [47] with recent gene annotations (Gencode v22) [48]. Differential expression was analyzed by DESeq2 [49]. Correlation between replicates and differential gene expression between time points was assessed by principal component analysis (PCA). RNA-Seq data have been deposited in the GEO under accession codes GSE85857. In addition, mRNA expression data was uploaded to IPA (www.ingenuity.com) and analyzed using default parameters. The expression heat map was generated using GENE-E (Broad Institute, MA, USA www.broadinstitute.org/cancer/software/GENE-E/). Fifty-eight EMT genes were selected by using the list from [50, 51].

### ChIP-qPCR

Runx1 ChIP-qPCR was performed essentially as described [52]. Briefly, 200,000 MCF10A cells were cross-linked, lysed and sonicated to obtain DNA fragments mostly in the 200-1000-bp range. Immunoprecipitation was performed at 4°C overnight with anti-Runx1 antibody (4334, Cell Signaling Technology) at a 1:15 antibody to chromatin ratio. Primers used in ChIP-qPCR are listed below: CDH1 Forward: CCCAACCTGACCACAGGAAT, CDH1 Reverse: GCTGCATGCGTAACAACACA; TGFB2 Forward: AGTCCTCCTCCCCCTAATGT, TGFB2 Reverse: CAGGGTATAGGCCACGACTG; TGFBR3 Forward: TCTTTGTAGCCTGCTGGGTT, TGFBR3 Reverse: CCCCCATCCTTACAAGTGGTT; ZNF333 (negative control 1) Forward: TGAAGACACATCTGCGAACC, ZNF333 Reverse: TCGCGCACTCATACAGTTTC; ZNF180 (negative control 2) Forward: TGATGCACAATAAGTCGAGCA, ZNF180 Reverse: TGCAGTCAATGTGGGAAGTC.

### Statistical analysis

The results were reported as Mean ± S.E.M. unless otherwise indicated, and Student's t-Tests were used to calculate statistical significance.

The following datasets were generated:

RNA-sequences: http://www.ncbi.nlm.nih.gov/geo/query/acc.cgi?acc=GSE85857, publicly available at NCBI Gene Expression Omnibus (accession no. GSE 85857).

## SUPPLEMENTARY MATERIALS FIGURES


