# Supplementary material for: Runx1 stabilizes the mammary epithelial cell phenotype and prevents epithelial to mesenchymal transition
Source: Oncotarget. 2017 Feb 16;8(11):17610–27. doi: 10.18632/oncotarget.15381 (PMC5392273; doi:10.18632/oncotarget.15381)
Supplement: Supplementary file 1 [file oncotarget-08-17610-s001.pdf]

Runx1 stabilizes the mammary epithelial cell phenotype and prevents epithelial to mesenchymal transition

SUPPLEMENTARY FIGURES

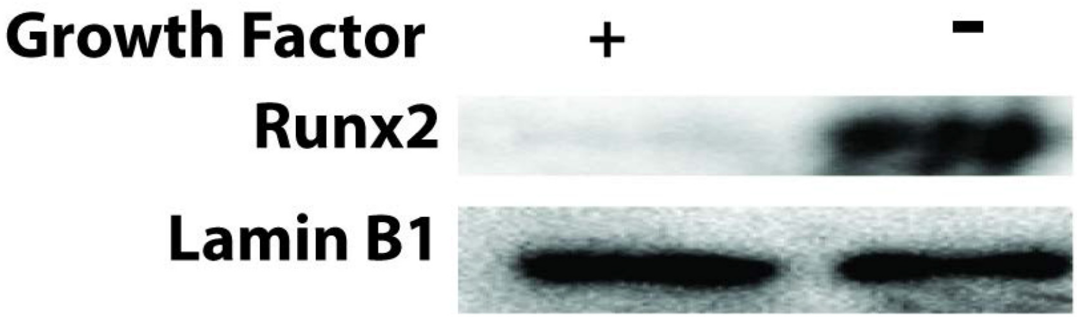

Supplementary Figure 1: Increased Runx2 during growth factor depleted induced EMT. Western blot analyses of cell lysates from MCF10A cells treated with or without growth factors showing changes in Runx2 activation during EMT.

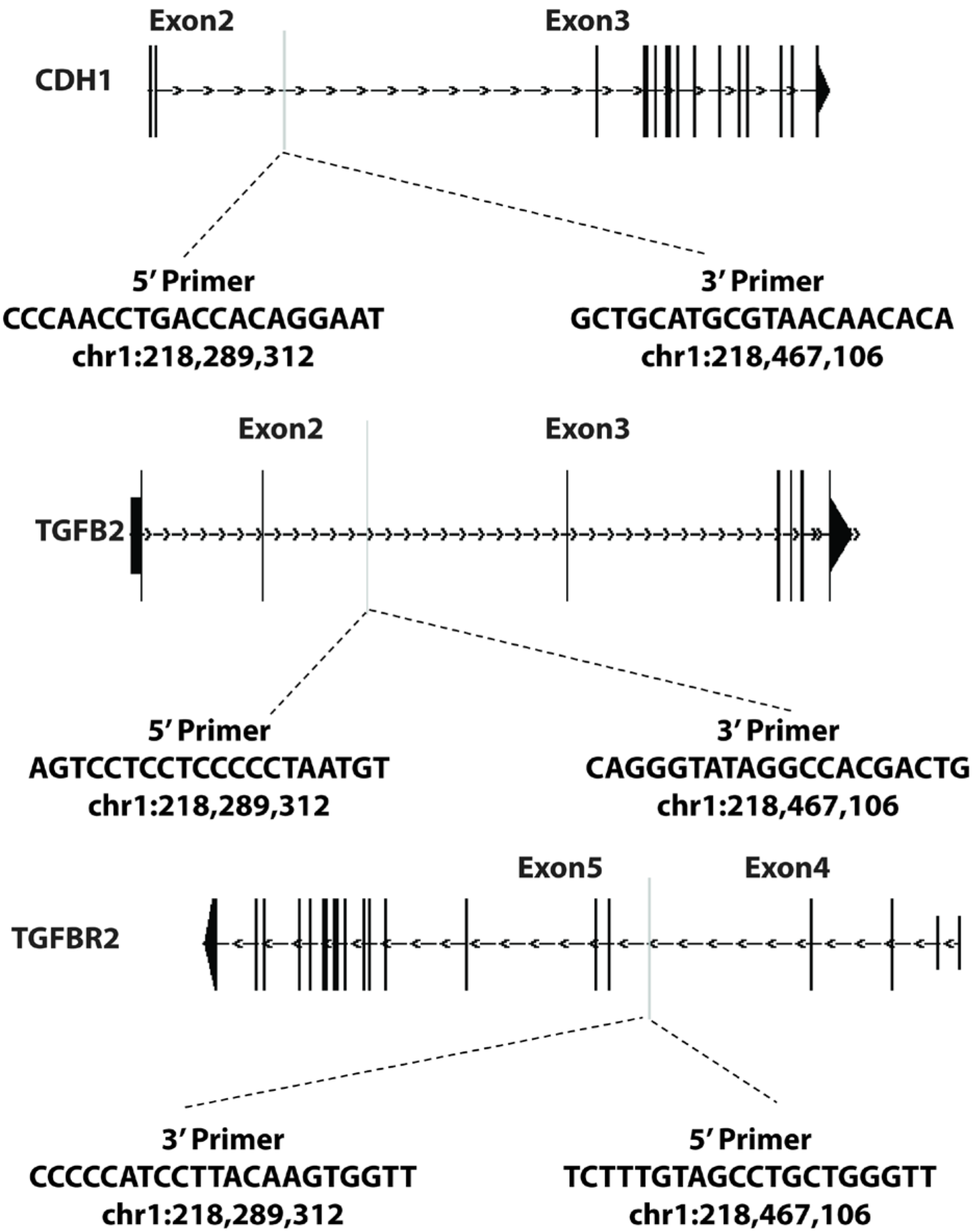

Supplementary Figure 2: Schematic diagram of ChIP qPCR primers and amplicons over the tested gene for ChIP-qPCR.

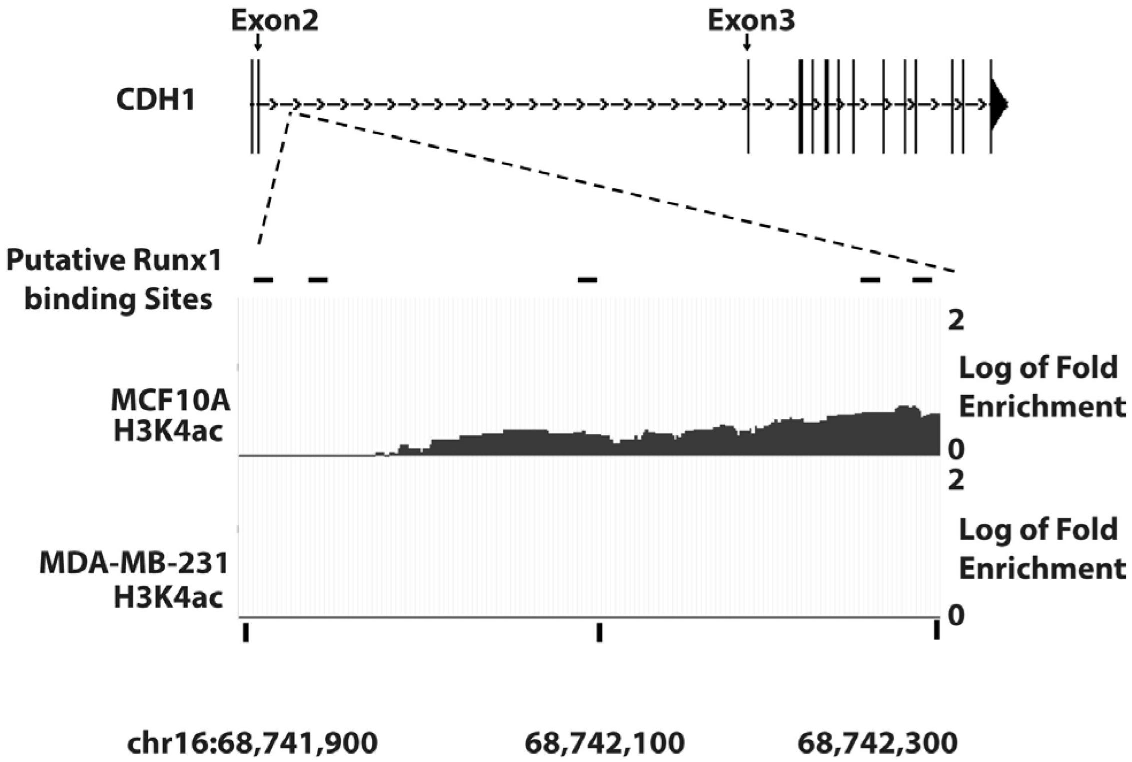

Supplementary Figure 3: Runx1 consensus sequences in CDH1 are coincident with H3K4Ac peaks in MCF10A cells. ChIP analysis showing significant binding of H3K4Ac (GES#) to a region in CDH1 genes with multiple Runx1 binding motifs in MCF10A cells but not in MDA-MB-231 cells.

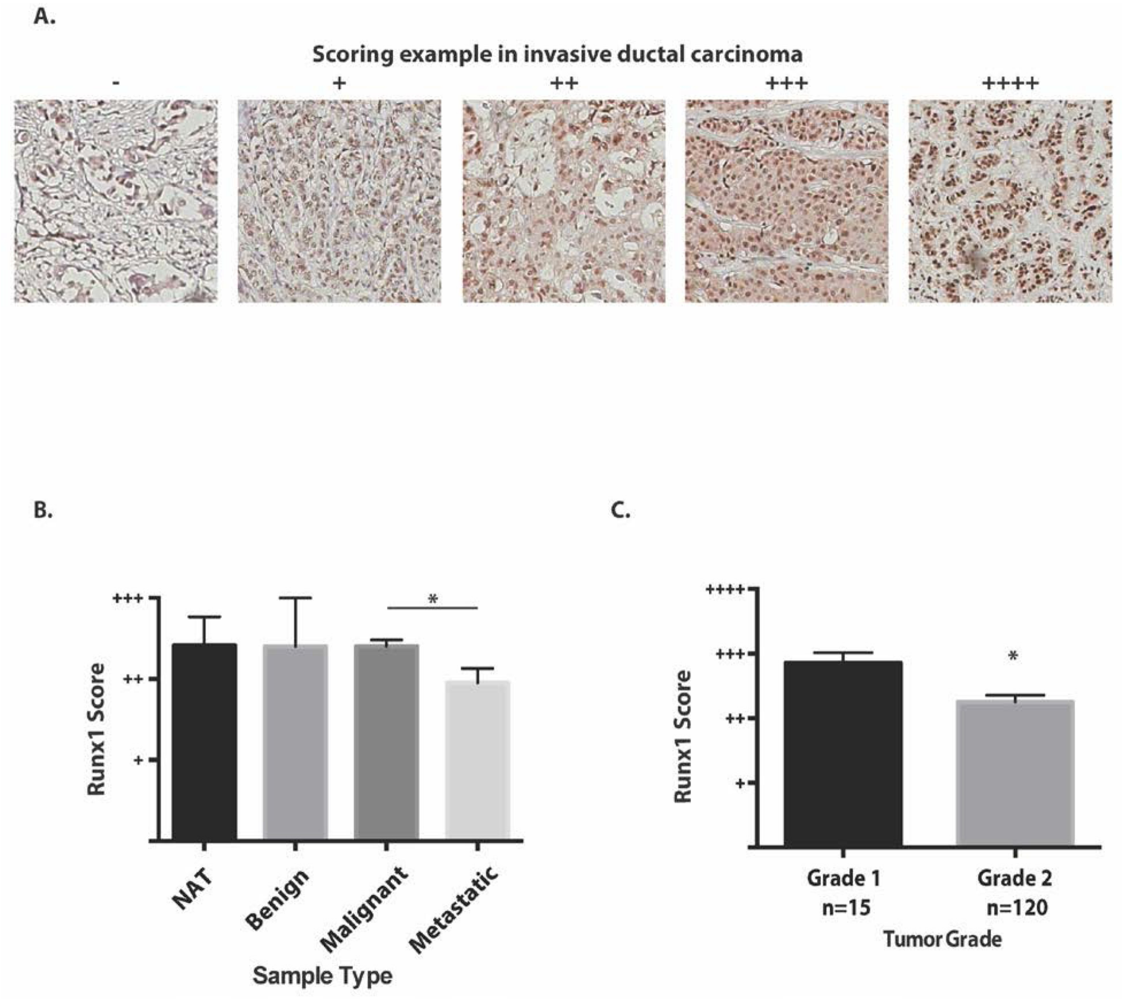

Supplementary Figure 4: Runx1 tissue microarray show that Runx1 is associated with early stage tumor. A. Representative tissue microarray images of Runx1 in invasive ductal carcinoma represent each scoring. **B.** Runx1 in scoring each category including normal adjacent tissue (NAT), fibroadenoma, invasive ductal carcinoma, and tumor metastasis to lymph. **C.** Runx1 scoring in grade 1 and grade 2 tumors.
